# Efficacy of second-line chemotherapy or immune checkpoint inhibitors for patients with a prolonged objective response (≥ 6 months) after first-line therapy for recurrent or metastatic head and neck squamous cell carcinoma: a retrospective study

**DOI:** 10.1186/s12885-023-11133-5

**Published:** 2023-07-14

**Authors:** Agathe Vienne, Laetitia Collet, Thomas Chevalier, Christian Borel, Magalie Tardy, Florence Huguet, Sandrine Richard, Sebastien Salas, Esma Saada-Bouzid, Jerome Fayette, Amaury Daste

**Affiliations:** 1 Department of Medical Oncology, Hôpital Saint-André, CHU Bordeaux-University of Bordeaux, 1 Rue Jean Burguet, Bordeaux, 33000 France; 2 Department of Medical Oncology, Léon Bérard Center, University of Lyon, Lyon, France; 3 Breast Cancer Translational Research Laboratory, Institut Jules Bordet, Bruxelles, Belgium; 4 Department of Medical Oncology, CHU la Timone, AP-HM, Marseille, France; 5 Department of Medical Oncology, Institut de Cancérologie Strasbourg Europe, Strasbourg, France; 6 Department of Medical Oncology, Centre Antoine Lacassagne, Université Côte d’Azur, Nice, France; 7 Department of Radiation Oncology, AP-HP, Tenon Hospital, Sorbonne University, Paris, France; 8 Department of Medical Oncology, Tenon Hospital, AP-HP Sorbonne University, Paris, France

**Keywords:** Platinum chemotherapy, Head and neck, Immune checkpoint inhibitors, Diseases free interval

## Abstract

**Background:**

Patients with recurrent or metastatic head and neck squamous cell carcinoma (R/M-HNSCC) have a poor prognosis and limited therapeutic options. Immune checkpoint inhibitors (ICIs) are effective in patients with tumor progression < 6 months following first-line, platinum-based chemotherapy (PBC), but data are missing for patients with progression ≥ 6 months after the last platinum dose.

**Methods:**

Retrospective analysis (six French centers, 2008–2019) of all consecutive R/M-HNSCC patients. treated first-line with PBC and tumor progression ≥ 6 months after the last platinum dose. Primary endpoint: progression-free survival after second-line therapy (PFS2). Additional endpoints: overall survival from Day 1 of first-line (OS1) and second-line (OS2) therapy.

**Results:**

R/M-HNSCC patients (n = 144) received cisplatinum (n = 67, 47%) or carboplatinum (n = 77, 53%) first-line. Response after first-line: complete response (CR; n = 16, 11%); partial response (PR; n = 77, 53%); stable disease (n = 22, 15%). Second-line therapy: PBC (n = 95, 66%); platinum-free regimen (PFR) (n = 25, 17%); ICI (n = 24, 17%). Median [95% confidence interval] PFS (months): PBC 5.0 [3.8–6.2]; PFR 4.0 [1–7.0]; ICI 2.0 [0.4–3.6] (p = 0.16). For PBC, PFR, and ICI, respectively: OS1 30, 23, and 29 months (p = 1.02); OS2 14, 10, and 16 months (p = 0.25); PR, 26%, 16%, and 21% patients; CR, 0%, 8%, and 4% patients. For subsequent lines, ICIs were administered for PBC (n = 11, 12%) and PFR (n = 2, 8%). No predictive factor for efficacy (PFS, OS) was identified.

**Conclusions:**

Our retrospective study suggests similar efficacy regarding OS2 for second-line chemotherapy or ICI in R/M-HNSCC patients with progression ≥ 6 months after the last first-line platinum dose.

## Introduction

More than 50% patients with locally-advanced head and neck squamous cell carcinomas (HNSCC) have a relapse within three years, despite multimodal treatments [1]. Until recently, most received platinum and cetuximab-based chemotherapy as first-line treatment for recurrent/metastatic (R/M-) HNSCC, such as the EXTREME (platinum, 5FU, cetuximab [2]) or TPEX (platinum, docetaxel, cetuximab [3]) regimens. For both regimens, maintenance of cetuximab is recommended until progression or unacceptable toxicity.

Limited therapeutic options are available for second-line therapy. After platinum failure, immune checkpoint inhibitors (ICI) have shown benefits regarding overall survival (OS) for patients with tumor progression < 6 months after the last dose of platinum compared with a single-agent therapy, leading to the approval of monotherapy with nivolumab for in R/M-HNSCC platinum-refractory patients or pembrolizumab in the same situation when the programmed cell death ligand-1 (PDL-1) tumor proportion score is > 50% [4, 5]. In patients who have progressed ≥ 6 months after the last dose of first-line platinum, there are currently no recommendations regarding whether to choose rechallenge using a platinum-based regimen, another chemotherapy, or ICI.

Accordingly, we performed a retrospective analysis in six French centers to evaluate the progression-free survival (PFS; primary endpoint) and overall survival (OS) in patients with R/M-HNSCC with tumor progression ≥ 6 months after the last dose of their first-line, platinum-based chemotherapy (PBC) and were treated second-line using either a new PBC, a platinum-free regimen (PFR), or an ICI.

## Methods

### Study design and patients

Our retrospective study was performed in six French centers of oncology (CHU de Bordeaux, Centre Léon Berard Lyon, CHU La Timone Marseille, Institut de Cancérologie Strasbourg Europe, Centre Antoine Lacassagne Nice, Hôpital Tenon AP-H Paris). Eligible patients (treated between 2008 and 2019) had histologically-confirmed R/M-HNSCC of the oral cavity, pharynx, or larynx that was not amenable to curative treatment, tumor recurrence or progression ≥ 6 months after the last dose of first-line PBC, and received second-line therapy with a new PBC, PFR, or ICI. The study was conducted in accordance with the criteria of practical clinical research.

### Statistical analysis

The primary endpoint was PFS after second-line therapy (PFS2), defined as the time between the start of the second-line treatment and clinical or radiological progression. Secondary endpoints were overall survival (OS), objective response rate (ORR) after second-line therapy (including complete response (CR), partial response (PR), and stable disease (SD)), and safety. Overall survival was defined as the time between the start (Day 1) of first-line (OS1) or second-line therapy (OS2) and death. PFS1 was defined as the time between the start of first-line therapy and radiological progression or death. All statistics are descriptive. Both PFS and OS were estimated using the Kaplan-Meier method.

## Results

A total of 144 patients were included. At the initial presentation, 16 patients (11%) had a stage IV tumor. The mean time between the end of initial treatment for locally-advanced disease and first relapse was 33 months [0-283 months].

As first-line therapy for R/M-HNSCC, most patients received a combination of platinum and anti-EGFR treatment (n = 131, 91%). Regimens included EXTREME (n = 95, 66%), TPEX (n = 15, 10%), or platinum with cetuximab (n = 16, 11%). A total of 77 patients (53%) received carboplatinum and 67 (47%) received cisplatinum. Complete response (CR) and partial response (PR) were obtained for 16 patients (11%) and 77 patients (53%), respectively.

Patient characteristics at second-line treatment are presented in Table 1. Second-line therapy comprised a new PBC in 95 patients (66%), mainly with carboplatinum (n = 69, 72%); of these, anti-EGFR was prescribed to 85 patients (89%), while FU and taxane were associated with platinum in 37 patients (39%) and 22 patients (23%), respectively. Twenty-five patients (17%) received PFR second-line; monochemotherapy without targeted therapy was administered to 12 patients (48%) (paclitaxel n = 5, docetaxel n = 1, methotrexate n = 5, vinorelbine n = 1) and polychemotherapy (methotrexate and vinflunine) to one patient (4%); anti-EGFR was only used with monochemotherapy and associated with paclitaxel (n = 12, 48%). ICI was prescribed to 24 patients (17%) second-line, including combination with anti CTLA-4 in five patients (5%).

**Table 1:** Patient characteristics at initiation of second-line therapy

		Platinum-based chemotherapy		Platinum-free regimen		Immune checkpoint inhibitors		Total	
		N	(%)	N	(%)	N	(%)	N	(%)
Total		95	(66)	25	(17)	24	(17)	144	(100)
Sex	F	21	(22.1)	6	(24.0)	2	(8.3)	29	(20.1)
	M	74	(77.9)	19	(76.0)	22	(91.7)	115	(79.9)
Tobacco	No	14	(15.4)	2	(8.7)	1	(4.2)	17	(12.3)
	Yes	50	(54.9)	12	(52.2)	10	(41.7)	72	(52.2)
	Ex	27	(29.7)	9	(39.1)	13	(54.2)	49	(35.5)
Alcohol	No	35	(36.8)	9	(36.0)	13	(54.2)	57	(39.6)
	> 30 gr	53	(55.8)	13	(52.0)	11	(45.8)	77	(53.5)
Localization	Oropharynx	34	(35.8)	3	(12.0)	3	(12.5)	40	(27.8)
	Hypopharynx	17	(17.9)	7	(28.0)	8	(33.3)	32	(22.2)
	Larynx	16	(16.8)	3	(12.0)	5	(20.8)	24	(16.7)
	Oral cavity	28	(29.5)	12	(48.0)	8	(33.3)	48	(33.3)
HPV 16	No	13	(13.7)	4	(16.0)	5	(20.8)	22	(15.3)
	Yes	3	(3.2)	1	(4.0)	2	(8.3)	6	(4.2)
	ND	79	(83.2)	20	(80.0)	17	(70.8)	116	(80.6)
Type of recurrence	Locoregional	51	(53.7)	13	(52.0)	13	(54.2)	77	(53.5)
	Metastatic	34	(35.8)	9	(36.0)	7	(29.2)	50	(34.7)
	Not recorded	10	(10.5)	3	(12.0)	4	(16.7)	17	(11.8)
Performance status ECOG2	0	17	(17.9)	3	(12.0)	5	(20.8)	25	(17.4)
	1	50	(52.6)	19	(76.0)	19	(79.2)	88	(61.1)
	≥ 2	25	(26.3)	3	(12.0)	0	(0.0)	28	(19.4)
	NR	3	(3.2)	0	(0.0)	0	(0.0)	3	(2.1)

**Fig. 1:**
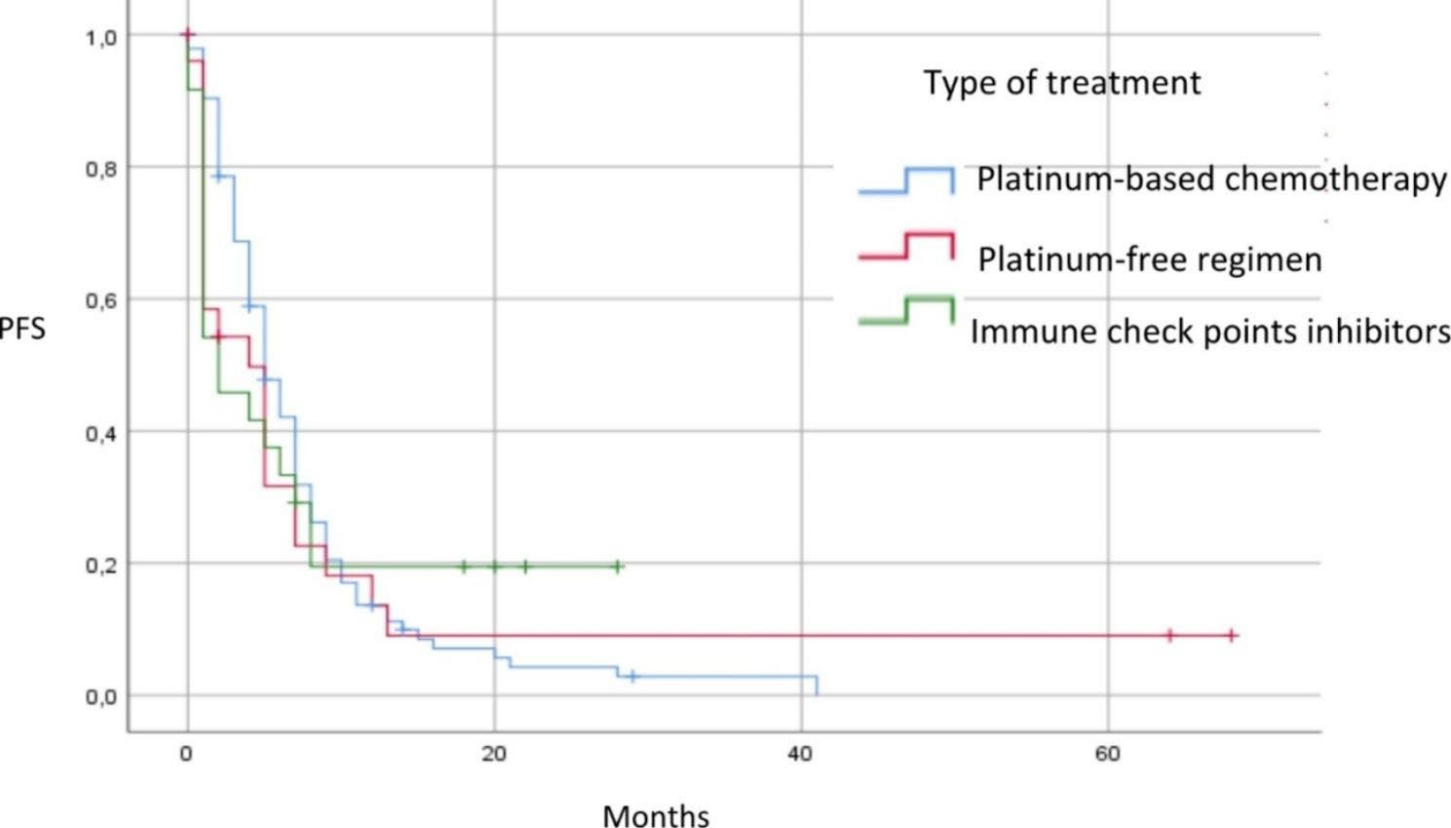
Kaplan-Meier estimate of progression-free survival (PFS) after second-line treatment

The median [95% confidence interval (CI)] PFS2 (in months) was 5 [4.13–5.87] for the entire cohort, and 5 [3.8–6.2], 4 [0.99–7.01], and 2 [0.4–3.6] for the PBC, PFR, and ICI cohorts, respectively (p = 0.92) (Fig. 1).

**Fig. 2:**
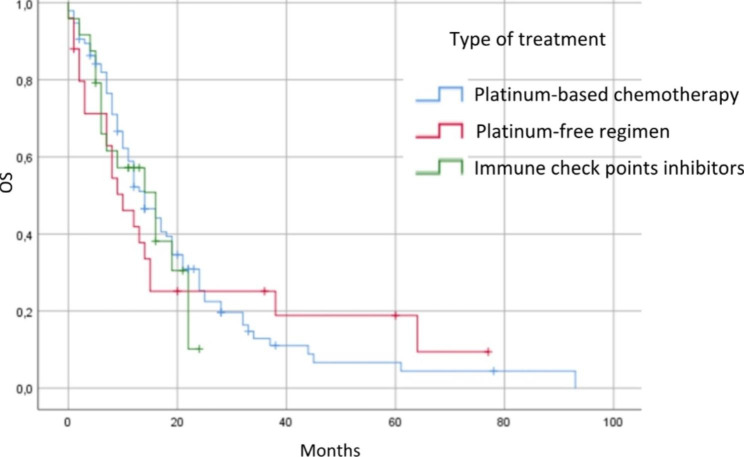
Kaplan-Meier estimate of overall survival (OS) after second-line treatment

The median [95% CI] OS2 (in months) was 13 [10.82–15.175] for the entire cohort, and 14 [10.5–17.5], 10 [5.2–14.8], and 16 [7.7–24.1] for the PBC, PFR, and ICI cohorts, respectively (p = 0.884) (Fig. 2).

For the PBC, PFR, and ICI cohorts, respectively: ORR occurred in 26%, 24%, and 25% of patients; CR occurred in 8% (PFR) and 4% (ICI) of patients, but was not evaluable for 29% (PBC) and 20% (PFR); SD was observed in 24%, 12%, and 17% of patients; progression was observed for 21%, 44%, and 54% of patients (Table 2).

**Table 2:** Objective response rates according to second-line treatment

	Platinum-based chemotherapy, N (%)	Platinum-free regimen, N (%)	Immune checkpoint inhibitors, N (%)
**Tumor response**			
CR/PR	25 (26)	6 (24)	6 (25)
SD	23 (24)	3 (12)	4 (17)
PD	20 (21)	11 (44)	13 (54)
UK	27 (29)	5 (20)	1 (4)

CR, complete response; PD, progressive disease; PR, partial response; SD, stable disease; UK, unknown

The median [95% CI] OS1 (in months) was 28 [24.1–31.8], and was similar between the different cohorts: PBC 30 [23.3–36.7], PFR 23 [16.1–29.9], and ICI 29 [18.7–39.3] (p = 0.6).

The safety profile was as expected for chemotherapy and ICI, with no new described events. More treatment discontinuations were reported in the PBC cohort (n = 9) than in the PFR cohort (n = 1). The main adverse event-related discontinuations were hematologic toxicity (n = 4) and cutaneous toxicity (n = 4). Only one patient stopped ICI for toxicity (myalgia).

In the PBC arm, there was no difference in efficacy regarding PFS or OS between patients who received taxane or 5FU or those who received a cisplatinum or carboplatinum regimen. Patients who received anti-EGFR with chemotherapy had a better median OS2 than patients without, but the difference was not statistically significant (p = 0.352).

## Discussion

Before the approval of ICI, patients with R/M-HNSCC after failure of first-line platinum therapy had a poor outcome, with no standard treatment for second-line treatment (best supportive care, methotrexate, paclitaxel, docetaxel, or cetuximab).

In our study, we analyzed 144 patients treated second-line at least 6 months after the last dose of first-line platinum therapy. Of these, 120 patients received chemotherapy +/- anti-EGFR. No statistically significant difference in PFS2 was seen between patients who received chemotherapy with or without platinum, while PFS2 was longer in the chemotherapy arms compared to ICI. Patients who received ICI had a similar median PFS to patients treated in the pivotal studies with anti-PD-1, nivolumab or pembrolizumab [4, 5]. In the EAGLE trial [6], which evaluated durvalumab (anti-PDL-1) alone or in combination with tremelimumab (anti-CTLA4) after a platinum regimen in R/M-HNSCC, patients could be included second-line six months after the last dose of platinum (in contrast to the CheckMate 141 [4] or KEYNOTE 040 [5] trials). In this phase 3 trial, the primary endpoint was not reached and the outcome in patients receiving the standard treatment was better than expected, with a median PFS of 2.1 and 3.7 months for the durvalumab and standard of care arms, respectively [6]. One hypothesis could be the greater efficacy of chemotherapy in patients who relapsed more than 6 months after the last first-line platinum dose, but these patients represented only 12.2% (n = 29) in the standard arm.

An option in second-line treatment remains re-challenge using the chemotherapy used first-line, after a drug-free interval, as reported for several cancers [7]. Disease progression after a time without therapy is not necessarily considered related to drug resistance, but could reflect a transient effect of the drugs. Due to the similar efficacy in patients treated with or without platinum-based chemotherapy in our cohorts, this suggests that patients with a progression-free interval (PFI) > 6 months after the last dose of platinum could be not only platinum sensitive but “chemotherapy sensitive”.

In our study, patients with recurrence ≥ 6 months after the last dose of platinum had a better outcome for median OS1 and OS2 than reported in other studies [2, 3]. This finding has also been seen in other tumors, such as ovarian cancer with an increase in OS when the PFI was > 6 months [8]. In R/M-HNSCC, there is no consensus on PFI from the last dose of platinum and recurrence regarding when to consider patients as platinum sensitive. In the literature, patients are commonly accepted to be platinum-refractory if progression occurs within 6 months after the last dose of platinum. However, Borel et al. described a similar response between patients with a PFI between 3 and 6 months and > 6 months in R/M-HNSCC patients [9].

In our cohort, no difference was seen in ORR and median PFS2 between patients who received mono- or polychemotherapy. However, there were more progressions in the PFR and ICI arms. This supports the concept that polychemotherapy and a rechallenge platinum regimen could be an option if a rapid response is necessary; however, if treatment tolerance is more important, without need for a rapid response, monochemotherapy could be proposed. Interestingly, patients in our cohort treated with cetuximab had a slighter better efficacy than patients with no anti-EGFR. It was probably important to continue this targeted therapy even in cases of progression with other chemotherapy regimens. Chevalier et al. [10] showed increased ORR when paclitaxel was associated with cetuximab versus paclitaxel alone after platinum failure. However, in this retrospective cohort the median PFS was similar in patients treated with and without cetuximab. Cetuximab was the only targeted therapy approved for first-line therapy in R/M-HNSCC [2, 3], but there are no data regarding the use of continuous cetuximab for subsequent lines.

Recently, pembrolizumab was approved first-line in R/M-HNSCC, alone or associated with platinum and 5FU [11]. However, after using a regimen combining platinum, FU, and pembrolizumab, the question of optimal treatment for second-line therapy became crucial. Some data have suggested increasing efficacy of chemotherapy after ICI [12, 13]. In our cohort, there was no difference in efficacy between second-line regimens for patients progressing 6 months after the last dose of first-line platinum.

Our study has some limitations. It is a retrospective study, although we analyzed all consecutive patients with a long response to platinum treatment for R/M-HNSCC. Due to the small numbers of patients, the period of analysis was long (2008 to 2019); however, the arrival of ICI represented a major change in practice during this period [2, 11]. During our period of analysis, ICI had only been administrated in clinical trials, which could be a patient selection bias and might explain the slight increase in median OS2 observed in our study. The profile of patients was probably different between the PBC and PFR arms. The physicians do not explain the choice of PBC or PFR. In the two groups, there was more monochemotherapy in the PFR cohort (probably due to comorbidities), which might explain the lower median OS compared with PBC. Moreover, the numbers of patients was lower in the PFR and ICI arms compared with the PBC cohort.

In conclusion, our study showed no difference in efficacy between second-line chemotherapy with or without platinum and ICI in R/M-HNSCC patients who had a relapse ≥ 6 months after the last dose of first-line platinum. Considering OS1 and OS2, patients exposed to PBC or ICI seemed to have a better outcome than those treated by chemotherapy without platinum.

## Data Availability

The datasets used and/or analyzed in the current study are available from the corresponding authors on reasonable request.
